# Immunohistochemical staining with non-phospho β-catenin as a diagnostic and prognostic tool of COX-2 inhibitor therapy for patients with extra-peritoneal desmoid-type fibromatosis

**DOI:** 10.1186/s13000-017-0654-z

**Published:** 2017-08-29

**Authors:** Tomohisa Sakai, Yoshihiro Nishida, Shunsuke Hamada, Hiroshi Koike, Kunihiro Ikuta, Takehiro Ota, Naoki Ishiguro

**Affiliations:** Department of Orthopaedic Surgery, Nagoya University Graduate School and School of Medicine, 65 Tsurumai, Showa, Nagoya, Aichi 466-8550 Japan

**Keywords:** Non-phospho β-catenin, Desmoid-type fibromatosis, Meloxicam, Prognosis, Diagnosis

## Abstract

**Background:**

Immunohistochemical staining with conventional anti-β-catenin antibody has been applied as a diagnostic tool for desmoid-type fibromatosis (DF). This study aimed to evaluate the diagnostic and prognostic value of immunohistochemical staining with anti-non-phospho β-catenin antibody, which might more accurately reflect the aggressiveness of DF, in comparison to the conventional anti-β-catenin antibody.

**Methods:**

Between 2003 and 2015, 40 patients with extra-peritoneal sporadic DF were prospectively treated with meloxicam or celecoxib, a COX-2 inhibitor, therapy. The efficacy of this treatment was evaluated according to Response Evaluation Criteria in Solid Tumors (RECIST). Immunohistochemical staining was performed on formalin-fixed material to evaluate the expression of β-catenin and non-phospho β-catenin, and the positivity was grouped as negative, weak, moderate, and strong. DNA was isolated from frozen tissue or formalin-fixed materials, and the *CTNNB1* mutation status was determined by direct sequencing.

**Results:**

Of the 40 patients receiving COX-2 inhibitor treatment, there was one with complete remission, 12 with partial remission, 7 with stable disease, and 20 with progressive disease. The mutation sites in *CTNNB1* were detected in 22 (55%) of the 40 cases: T41A (17 cases), S45F (3 cases), and T41I and S45P (1 each). The positive nuclear expression of non-phospho β-catenin showed a significant correlation with positive *CTNNB1* mutation status detected by Sanger method (*p* = 0.025), and poor outcome in COX-2 inhibitor therapy (*p* = 0.022). In contrast, nuclear expression of β-catenin did not show a significant correlation with either *CTNNB1* mutation status (*p* = 0.43) or outcome of COX-2 inhibitor therapy (*p* = 0.38).

**Conclusions:**

Nuclear expression of non-phospho β-catenin might more appropriately reflect the biological behavior of DF, and immunohistochemical staining with non-phospho β-catenin could serve as a more useful diagnostic and prognostic tool of COX-2 inhibitor therapy for patients with DF.

## Background

Desmoid-type fibromatosis (DF), also known as aggressive fibromatosis, is characterized by benign and locally infiltrative fibroblastic tumors. Extra-peritoneal DF are at high risk of local recurrence after planned surgery even with a wide surgical margin (range 14.1–68%) [1–5], and surgical treatment occasionally leads to crucial post-operative morbidity such as amputation or serious functional impairment such as severe limitation of the range of motion of an involved joint. On the other hand, some cases of DF show stabilization or spontaneous regression of tumor without treatment [6]. Considering these enigmatic behaviors, the therapeutic approach for extra-peritoneal DF has been shifting from surgery with a wide surgical margin to conservative therapy [7, 8]. Recently, several therapeutic modalities for DF were reported including ‘wait & see’ only [9], COX-2 inhibitor therapy [10], hormonal therapy [11], low-dose chemotherapy [12], and tyrosine kinase inhibitors [13, 14], while few studies have investigated the prognosticators of these conservative therapies thus far.

In the tumorigenesis of DF, activation of Wnt signaling plays an important role, and aberrant nuclear accumulation of β-catenin has been utilized for pathological diagnosis to differentiate DF from other conditions [15]. Somatic mutations at exon 3 of Catenin β-1 (*CTNNB1*) gene have been reported in 64–85% of sporadic DF. The mutation generally occurs at codon 41 or 45, with T41A (threonine to alanine), S45F (serine to phenylalanine), or S45P (serine to proline) being the most frequent [16–18]. These mutations inhibit phosphorylation of β-catenin, which protect β-catenin from degradation by APC (adenomatous polyposis coli) complex, resulting in nuclear accumulation of β-catenin, where it binds to the TCF/LEF family of transcription factors and turns on a number of target genes [19]. Fewer patients have mutations in Adenomatous Polyposis Coli (*APC*) sporadically. Mutation of *APC* gene is implicated in the pathogenesis of familial adenomatous polyposis (FAP). Functional impairment of APC complex is induced by these mutations, and β-catenin retains its non-phosphorylated status, translocates and accumulates in the nucleus. In patients with FAP, similar activation of Wnt-β-catenin pathway will occur to that in patients with *CTNNB1* mutation. The degree of β-catenin phosphorylation will differ based on the mutation type; codon 41, 45, wild type (WT), or *APC* gene. Together, the degree of Wnt-β-catenin pathway activation will be dependent on the mutation status of patients with DF. Recent studies have demonstrated that the mutation status of *CTNNB1* can help to predict the outcome of surgical [16, 17] and conservative therapies [18, 20]. In the clinical setting, immunohistochemical evaluation has been the standard for evaluation, and *CTNNB1* mutation cannot be evaluated at all institutions. A more appropriate antibody that better reflects desmoid biology is required for pathologists and physicians.

We previously reported that higher nuclear expression of β-catenin correlated with the efficacy of meloxicam treatment for patients with extra-peritoneal sporadic DF [21]. However, an increasing number of DF patients with lower nuclear β-catenin expression have proven resistant to meloxicam treatment since the previous report, prompting us to re-investigate the significance of nuclear β-catenin expression as a prognosticator for meloxicam treatment. Moreover, we hypothesized that non-phospho (active) β-catenin would reflect *CTNNB1* mutation status, and help to predict the treatment outcome of patients with DF more precisely than β-catenin.

## Methods

### Patients and outcome evaluation

Fifty cases with extra-peritoneal DF were diagnosed in our institutions since 2003. Ten cases were excluded from this study. Five of them did not agree to treatment with COX-2 inhibitor, or had already received “wait and see” follow-up before the referral with status of stable disease. In three, biopsy specimens and formalin-fixed histological preparations were not available for the analyses, while two were excluded due to lack of adequate imaging for evaluation. Finally, this study focused on 40 patients with extra-peritoneal DF, all of whom were prospectively observed with COX-2 inhibitor therapy (meloxicam in 38 and celecoxib in 2). There were no patients with definitive FAP-related DF based on the complete history examinaton. All 40 cases were histologically evaluated and diagnosed with DF by experienced pathologists after thorough discussion. At the beginning of treatment, DF was evaluated with magnetic resonance imaging (MRI) and/or computed tomography (CT) in all cases. Patients were examined physiologically and received MRI and/or CT on an outpatient basis every 3 to 6 months. The efficacy of COX-2 inhibitor treatment was determined based on Response Evaluation Criteria in Solid Tumors (RECIST) [22] evaluated with MRI or CT at the latest follow-up or the endpoint of COX-2 inhibitor therapy as compared to that at the beginning. Patients with PD status could discontinue this therapy and select other treatment options such as low-dose methotrexate and vinblastine therapy [10] or surgery [23] with careful consideration of the tumor features, which included location and infiltrative behavior of the tumor, and individual patient’s preference. Considering characteristics of DF (locally aggressive but no metastasis), SD status with no clinical symptom impairing patient’s QOL is thought to reflect tumor dormancy. Patients were classified into a unfavorable group (PD) and favorable group (CR, PR, SD).

### Mutation analysis of *CTNNB1*

Mutation analysis of *CTNNB1* was achieved as we previously described [18]. Briefly, DNA was extracted from frozen or formalin-fixed, paraffin-embedded (FFPE) tissue with the High Pure PCR Template Preparation Kit (Roche Molecular Diagnostics, Mannheim, Germany). DNA was amplified with polymerase chain reaction (PCR) (40 cycles, annealing temperature; 58 °C, extension temperature; 72 °C) using LightCycler 480 System (Roche). We used two pairs of primers to evaluate the presence or absence of point mutations in codons 41 or 45 of *CTNNB1* exon 3: forward 5′–GATTTGATGGAGTTGGACATGG–3′, reverse 5′- TCTTCCTCAGGATTGCCTT -3′, and forward 5′-TGGAACCAGACAGAAAAGCG-3′, reverse 5′- TCAGGATTGCCTTTACCACTC -3′. PCR products were extracted from the isolated bands after gel electrophoresis in 2% agarose, and purified. Direct sequencing was performed with the forward primers, using Applied Biosystems Big Dye Terminator V3.1, and Applied Biosystems 3730× DNA analyzer (Applied Biosystems, Foster City, CA) at FASMAC Co. Ltd. (Kanagawa, Japan). Results of the direct sequencing were evaluated using the databases of NCBI-BLAST to determine the mutation sites.

### Immunohistochemistry

Tumor specimens were obtained by needle or incisional biopsy in advance of COX-2 inhibitor therapy, fixed in 10% formalin, and embedded in paraffin, and subjected to immunohistochemical study to analyze the expression of β-catenin. According to the previous report [21], the slides were treated overnight at 4 °C with anti-human β-catenin mouse monoclonal antibody (M3539; Dako, Carpinteria, CA; 1:200 dilution) and anti-human non-phospho (active) β-catenin (Ser33/37/Thr41) rabbit monoclonal antibody (8814S; Cell Signaling Technology, Danvers, MA; 1:200 dilution), counterstained with hematoxylin, dehydrated, and mounted. Non-phospho (Active) β-Catenin rabbit monoclonal antibody recognizes endogenous β-catenin protein when residues Ser33, Ser37, and Thr41 are not phosphorylated. It does not detect β-catenin protein if tri-phosphorylated at Ser33/Ser37/Thr41. A previous report indicated that colon carcinoma was positively stained with this non-phospho β-catenin [24]. We used colon carcinoma tissues for non-phospho β-catenin staining as a positive control, and non-aggressive fibrous tumors, fibroma, was also subjected to this staining. This non-phospho β-catenin antibody was also used for western blotting using oral squamous cell carcinoma in another previous study [25]. Nuclear and cytoplasmic positivity of β-catenin and non-phospho β-catenin were analyzed by two observers (S. H., T. S.) without the clinical information of cases, and classified into 4 groups according to definition of a previous report [21]; 0% for positively stained cells (negative; 0), 1% to 10% (weak; 1+), 11% to 50% (moderate; 2+) and 51% to 100% (strong; 3+) on 10 randomly selected high-power fields. In accordance with a previous report [16], the intensity of nuclear staining was also evaluated. If positive staining was observed partially in cytoplasmic area adjacent to the positive nuclear staining, we evaluated the case as positive for both “nuclear” and “cytoplasmic”. Results of immunohistochemistry for β-catenin and non-phospho β-catenin were subjected to the association analyses between the positivity and efficacy of COX-2 inhibitor therapy or *CTNNB1* mutation status.

### Statistical evaluation

Fisher exact test and Pearson chi-square test were applied to examine correlations of di- or tri-chotomous variables among the outcome of COX-2 inhibitor therapy, clinical characteristics, results of nuclear staining for β-catenin and non-phospho β-catenin by immunohistochemistry, and *CTNNB1* mutation status. Continuous variables of age and tumor size were compared between the favorable and unfavorable groups, and highly positive and lower positive group for β-catenin and non-phospho β-catenin using the Mann-Whitney U test. All statistical analyses were performed using SPSS statistics 20 (IBM Corp. Armonk, NY). *P* < 0.05 was considered significant.

## Results

### Clinical features and outcome of COX-2 inhibitor therapy

Of the 40 patients with extra-peritoneal DF, 16 were male and 24 were female. The mean age was 41.7 years (median, 36.0 years; range, 10–87 years). The location of the tumor was the abdominal wall in 10 patients, other sites in the trunk in 11, extremities in 15, and neck in 4. The diameter of the tumor ranged from 22.5 to 143.7 mm (mean, 76.2 mm; median, 74.5 mm). There were no patients treated with radiotherapy or other conservative treatment including anti-hormonal therapy or low-dose chemotherapy of methotrexate and vinblastine for DF prior to the COX-2 inhibitor therapy.

Mean follow up duration from the first visit to the final follow up date of meloxicam therapy was 29.6 months ranging from 2 to 104 months. Of the 40 patients evaluated with RECIST criteria, one patient was classified with CR, 12 with PR, 7 with SD, and 20 with PD. Of the 20 cases with PD, 10 received surgical treatment, and 9 low-dose methotrexate with vinblastine and/or doxorubicin-based chemotherapy, while one refused any other treatment with only continuation of meloxicam therapy.

Between the favorable (CR, PR, and SD) and unfavorable (PD) groups, no variable was associated with a significant impact on the prognosis for COX-2 inhibitor therapy, Age (*p* = 0.089) and tumor size (*p* = 0.11) had a trend and marginal impact on the prognosis, respectively. Regarding the site of involvement, all 4 cases with neck involvement were assigned to the unfavorable group (Table 1).

**Table 1:** Patients characteristics between two prognosis group

Variables	Favorable group (*n* = 20)	Unfavorable group (*n* = 20)	*p* value
Gender			0.2
Female	10	14	
Male	10	6	
Mean age, years (range)	49.7 (19–87)	33.8 (10–74)	0.089
Mean size, mm (range)	70.6 (38.8–143.7)	81.8 (22.5–127.7)	0.11
Site			0.076
Abdominal wall	6	4	
Other trunk	4	7	
Extremities	10	5	
Neck	0	4	
*CTNNB1* mutation			0.2
T41A	8	9	
T41I	1	0	
S45F	0	3	
S45P	0	1	
Wild type	11	7	

### Mutation status of *CTNNB1*

All the 40 cases of the present study cohort received genotyping of *CTNNB1* exon 3. Point mutations existed in 22 of the 40 cases (55%) and were localized to just two codons (41 and 45). The most frequent mutation was replacement of threonine by alanine in codon 41 (T41A), which was detected in 17 cases (43%). Substitution of serine by phenylalanine in codon 45 (S45F) existed in 3 (8%), and that of threonine by isoleucine in codon 41 (T41I) and serine by proline in codon 45 (S45P) in one (3%) each. In the remaining 18 cases (45%), no mutation site was identified in the hot focus (codon 33–45) of *CTNNB1* gene (wild type) by Sanger method. Of interest all 4 cases with mutation in codon 45, including 3 cases with S45F mutation, showed progressive disease with COX-2 inhibitor therapy (Table 1).

### Immunohistochemical findings

**Fig. 1:**
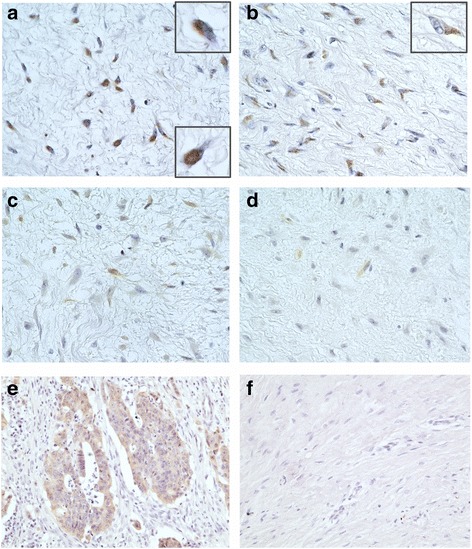
Immunohistochemical staining for β-catenin and non-phospho β-catenin Representative images were shown with staining for β-catenin (**a** and **b**) and non-phospho β-catenin (**c** and **d**). Nuclear and cytoplasmic staining was observed in **a** and **c** (a: upper right inset; higher magnification of cytoplasmic staining, a: lower right inset; higher magnification of nuclear staining). Only cytoplasmic staining was observed in b and d (b: upper right inset; higher magnification of cytoplasmic staining). Positive staining was observed in human colon carcinoma tissue (**e**). A representative case of fibroma of tendon sheath showed negative staining (**f**). (counterstained with hematoxylin; original magnification: **a**-**d**; ×400, e-f; ×200).

**Fig. 2:**
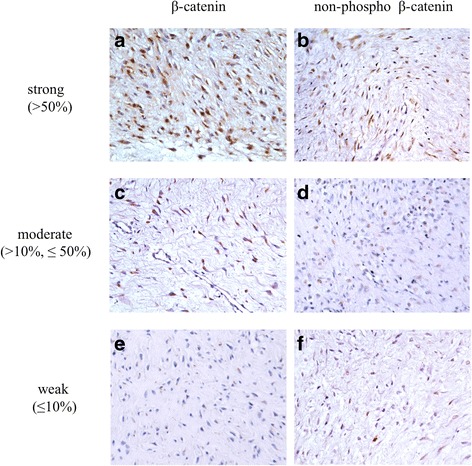
Nuclear staining for β-catenin and non-phospho β-catenin. Representative images were shown with staining for β-catenin (**a**, **c**, **e**) and non-phospho β-catenin (**b**, **d**, **f**). **a** and **b**; strong, **c** and **d**; moderate, **e** and **f**; weak. (counterstained with hematoxylin; original magnification, ×200)

In all 40 cases, positivities and intensities of nuclear β-catenin and non-phospho β-catenin staining were evaluated. Nuclear and/or cytoplasmic staining pattern was observed, which was dependent on each case (Fig. 1a-d). Colon carcinoma was positive as a control (Fig. 1e). Three cases of fibroma of tendon sheath were subjected to non-phospho β-catenin staining, and all cases showed negative stainability. A representative staining was shown in Fig. 1f. With evaluation of nuclear positivities for β-catenin staining, there was no case with negative staining status. 6 cases showed weak, 22 cases showed moderate, and 12 cases showed strongly positive staining. On the other hand, with evaluation of non-phospho β-catenin staining, 4 cases showed negative staining status. 21 cases showed weak, 13 cases showed moderate and 2 cases showed strongly positive staining (Fig. 2) (Table 2). In the evaluation of positivity for cytoplasmic staining, no case showed negative staining status for either β-catenin or non-phosphop β-catenin staining. Nineteen cases showed moderate and 21 cases showed strong β-catenin staining, and 21 cases showed weak, 13 cases moderate, and 6 cases strong staining for non-phosphop β-catenin.

**Table 2:** Nuclear expression of β-catenin and non-phospho β-catenin. Relationship with *CTNNB1* mutation status

	Mutation status					
	WT	T41A	T41I	S45F	S45P	*p* value
β-catenin^a^						0.43
weak	4	2	0	0	0	
moderate	8	12	1	1	0	
strong	6	3	0	2	1	
non-phospho β-catenin						0.025
negative	3	0	1	0	0	
weak	10	11	0	0	0	
moderate	4	6	0	2	1	
strong	1	0	0	1	0	

*WT* wild type

^a^No cases showed negative

As a result, in non-phospho β-catenin staining, all 40 cases showed the same degree or weaker staining status compared with that of β-catenin. It could be explained that anti-non-phospho β-catenin antibody did not detect β-catenin protein if tri-phosphorylated at Ser33/Ser37/Thr41, in contrast to anti-β-catenin antibody, which could detect β-catenin protein including the tri-phosphorylated type. With respect to nuclear staining intensity for β-catenin, there were 13 cases with weak, 16 with moderate and 11 with strong intensity. For non-phospho β-catenin, there were 4 cases with negative, 14 with weak, 17 with moderate and 5 with strong intensity.

### Correlation between mutation status of *CTNNB1* and immunohistochemical status of β-catenin

Positive nuclear staining of non-phospho β-catenin was significantly correlated with positive *CTNNB1* mutational status (*p* = 0.025) detected by Sanger sequencing, whereas no correlation was observed between nuclear staining of β-catenin and that of *CTNNB1* (*p* = 0.43). An interesting finding was that fewer cases (*n* = 1) showed strongly positive for non-phospho β-catenin in WT, T41A, and T41I as compared with that (*n* = 9) for β-catenin. Among cases with WT and T41A mutated cases, only 30% cases showed strong or moderate positivity for non-phospho β-catenin staining, whereas more than 80% cases showed strong or moderate for β-catenin staining. In contrast, all four cases with mutation of codon 45 (S45F and S45P) showed strong or moderate nuclear positive staining for both β-catenin and non-phospho β-catenin (Table 2).

### Correlation between clinical outcome of COX-2 inhibitor therapy and immunohistochemical results of β-catenin

Statistical analyses with Fisher’s exact test revealed that neither β-catenin nor non-phospho β-catenin significantly correlated with the clinical outcome of COX-2 inhibitor therapy (*p* = 0.16 and *p* = 0.11, respectively) (Table 3). Of the 12 cases with strong nuclear staining for β-catenin, 4 showed a favorable outcome and 8 an unfavorable one. Interestingly, both of the 2 cases with strong nuclear expression of non-phospho β-catenin were evaluated as unfavorable. These results prompted us to set the adequate cut off value for positivity of β-catenin staining. A cut off value of 10% (negative and weak vs moderate and strong) demonstrated that stronger nuclear expression of non-phospho β-catenin had a significant impact on prognosis (*p* = 0.022), whereas that of β-catenin expression did not (*p* = 0.38) (Table 4). On the other hand, cytoplasmic staining status of both β-catenin and non-phospho β-catenin staining had no significant correlation with the clinical outcome (*p* = 0.75 and *p* = 0.51, respectively). Nuclear staining intensity of β-catenin and non-phospho β-catenin had no significant correlation with clinical outcome (*p* = 0.91 and *p* = 0.67, respectively). Analyses of the relationship between staining positivity of non-phospho β-catenin and clinical variables revealed that age (*p* = 0.013) and tumor size (*p* = 0.081) had a correlation (Table 5).

**Table 3:** Nuclear expression of β-catenin and non-phospho β-catenin. Relationship with clinical outcome of COX-2 inhibitor therapy

	Favorable group (*n* = 20)	Unfavorable group (*n* = 20)	*p* value
β-catenin^a^			0.16
weak	2	4	
moderate	14	8	
strong	4	8	
non-phospho β-catenin			0.11
negative	3	1	
weak	13	8	
moderate	4	9	
strong	0	2	

^a^No cases showed negative

**Table 4:** Nuclear expression of β-catenin and non-phospho β-catenin on cut off value of 10% setting. Relationship with clinical outcome of COX-2 inhibitor therapy

	Favorable group (*n* = 20)	Unfavorable group (*n* = 20)	*p* value
β-catenin			0.38
≤ 10%	2	4	
> 10%	18	16	
non-phospho β-catenin			0.022
≤ 10%	16	9	
> 10%	4	11

**Table 5:** Clinical variables and nuclear positivity for non-phospho β-catenin

Variables	Nuclear non-phospho β-catenin staining		
	≤10% (*n* = 25)	>10% (*n* = 15)	*p* value
Gender			0.5
Female	14	10	
Male	11	5	
Mean age, years (range)	48.7 (18–87)	31.4 (10–67)	0.013
Mean size, mm (range)	70.3 (38.8–130.2)	86.0 (22.5–143.7)	0.081
Site			0.95
Abdminal wall	6	4	
Other trunk	7	4	
Extremities	9	6	
Neck	3	1	

## Discussion

Wnt signaling plays an important role in the tumorigenesis of DF [26]. In the absence of Wnt stimulation, β-catenin is continuously phosphorylated by a multiprotein complex composed of tumor suppressor proteins APC, Axin, casein kinase 1 (CK1), and glycogen synthase kinase-3β (GSK-3β). In this process, phosphorylation starts at the S45 of N terminus encoded by exon 3 of the *CTNNB1* gene by ‘priming kinase’ CK1, then at T41, S37, and S33 by GSK3β. Phosphorylation of β-catenin by the APC-axin-GSK-3β complex leads to its degradation by the ubiquitin-proteasome system. With a mutation of any one of these amino acid residues in exon 3 of the *CTNNB1* gene or the presence of Wnt stimulation, β-catenin retains non-phosphorylation status, accumulates and translocates into the nucleus, where it binds to the TCF/LEF family of transcription factors and turns on a number of target genes [19]. In shedding light on the underlying pathogenesis of DF, evaluation for phosphorylation status can serve as a biomarker of DF aggressiveness.

Several studies have reported the correlation between nuclear expression of β-catenin by immunohistochemical examination and the prognosis of patients with DF. Gebert et al. revealed that nuclear β-catenin expression was significantly associated with an increased rate of local recurrence after surgical resection (5-year event-free survival; 0%, *P* < 0.05) [27]. They used a tissue microarray of 37 cases, although clinical follow-up data were available in only 23 cases. Our previous study reported that higher nuclear expression of β-catenin was significantly associated with a poor response (*p* = 0.017) to meloxicam therapy based on the 31 cases investigated [21]. However, in additional analyses of the cases accumulated after the previous report [21], an increased number of cases showed resistance to COX-2 inhibitor therapy. This may explain the discrepancy in the results between the previous and present studies regarding the usefulness of nuclear expression of β-catenin as a prognostic marker for COX-2 inhibitor therapy (previous report; *p* = 0.017, present study, *p* = 0.38). Lazar et al. observed nuclear β-catenin expression in 98% of specimens, with the percentage of positive nuclear staining cells for β-catenin not correlated with desmoid recurrence, and intensity inversely correlated with the incidence of desmoid recurrence (*p* < 0.01) [16]. The reason why decreased nuclear β-catenin intensity was associated with a higher recurrence rate of desmoid was not clarified nor speculated on in their study. The present study using non-phospho β-catenin antibody may provide more useful information based on the mechanistic aspects because non-phospho β-catenin antibody detects β-catenin only when residues S33, S37 and T41 are not phosphorylated, that is active β-catenin. There were 5 cases with “wait and see” policy, which were not included in the present study. Three cases were unfavorable course and 2 favorable. Interestingly, 2 favorable cases showed weak or negative nuclear stainability for non-phospho β-catenin, 3 unfavorable cases showed strong (1), moderate (1), or weak (1).

Recently, as the treatment algorithm for extra-peritoneal DF has been shifting from primary wide resection to conservative therapy, prognosticators for conservative therapy are also urgently needed. The correlation between *CTNNB1* mutation status and clinical outcome of DF has been focused on in several reports. In surgical treatment, DF with the S45F had a greater tendency for local recurrence compared to DF without S45F mutation [16, 17, 28]. A poor clinical outcome in DF patients with S45F was also reported in COX-2 inhibitor therapy with meloxicam [21]. Consistent with these studies, DF with S45F mutation were all evaluated as belonging to the unfavorable group for meloxicam therapy in the present study. However, how the mutation is implicated in the poor clinical outcome observed has not been fully analyzed and clarified. We previously reported that cultured DF cells obtained from a patient with S45F mutation showed strong nuclear expression of β-catenin immunohistochemically and higher mRNA expression levels of Wnt target genes, Axin-2 and Cyclin-D1, with RT-PCR compared with DF cells from patients without S45F mutation (T41A and wild type) [29]. These findings indicate that DF with S45F mutation shows higher upregulation of Wnt signal, which may contribute to the poor clinical outcome seen with conservative treatment as well as surgery. Because convenient means for evaluation of prognosis are preferably applied in the clinical setting, immunohistochemical evaluation using non-phospho β-catenin will be more easily utilized than mutation analysis.

Certainly mutation S45F might be a prognosticator for treatment in DF, although the proportion of DF with S45F was only 6–28% of extra-peritoneal DF [16–18, 28, 30]. Although prognostic factors other than *CTNNB1* mutation have been reported such as age, size and location of the tumor [3, 15, 34, 35], a biomarker reflecting a more mechanistic property of desmoid is necessary for better evaluation of prognosis. In this study, non-phospho β-catenin staining could categorize tumors with S45F mutation detected by Sanger method as strong or moderate, and those with other mutations as strong-negative. Other factors may affect the stainability of the staining. Other than *CTNNB1* mutation, several causes of Wnt signal activation such as *APC* mutation [31], activation via TGF-β signal [32] and connective tissue growth factor (CTGF) have been reported [33].

There were several limitations in our study. First, the number of patients was small due to the rarity of DF, and thus it may have been underpowered to detect a significant difference between groups. However, the cohort of this study was prospectively treated with COX-2 inhibitor and subjected to *CTNNB1* mutation analysis. It is valuable and worth accumulating further studies. Second, non-phospho β-catenin staining could not completely predict the prognosis of patients with COX-2 inhibitor therapy. Nine of the 20 patients with an unfavorable outcome showed negative/weak nuclear non-phospho β-catenin expression, suggesting that Wnt signal activation is not the sole prognosticator in DF. Of clinical characteristics, younger age [34, 35] and larger tumor size [3, 34, 35] were reported as negative prognostic factors. In the future, a nomogram for prognosis should be established including clinical factors, *CTNNB1* mutation status, and results of immunohistochemical staining. Third, gene mutation was analyzed only in *CTNNB1* exon 3 by direct sequencing. Recently Crago et al. performed whole-exome sequence on wild type DF diagnosed with direct sequencing, and reported that 5 of 8 cases with wild type actually had the mutation in Wnt-related gene (3 in *CTNNB1*, 2 in *APC*) [36]. Similarly, our present study might also include some cases with wild type, which would harbor mutations of Wnt-related genes. Actually, mutation status is now investigated in different multi-center study using next generation sequencing, and preliminary results indicates one of the “wild type” cases in the present study has *APC* mutation despite no history of FAP. Another possible reason is that inadequate fixation time in formalin may cause low quality of extracted DNA, and the false negative results of *CTNNB1* mutation status because 22 cases were analyzed using FFPE samples. Further studies with additional molecular analyses will be required to better determine the correlation of prognosis and patient characteristics, mutation status, and immunohistochemical findings.

## Conclusions

We demonstrate for the first time that nuclear non-phospho β-catenin expression could predict the treatment efficacy of with COX-2 inhibitor in patients with sporadic DF. Larger prospective clinical studies and molecular analyses are still necessary to determine the significance of this non-phospho β-catenin staining.

## Abbreviations

APC: adenomatous polyposis coli
CK1: casein kinase 1
CT: computed tomography
CTGF: connective tissue growth factor
DF: desmoid-type fibromatosis
FAP: familial adenomatous polyposis
GSK-3β: glycogen synthase kinase-3β
MRI: magnetic resonance imaging
PCR: polymerase chain reaction
RECIST: response evaluation criteria in solid tumors
WT: wild type
